# Mechanisms Underlying the Link between Cannabis Use and Prospective Memory

**DOI:** 10.1371/journal.pone.0036820

**Published:** 2012-05-11

**Authors:** Carrie Cuttler, Ryan J. McLaughlin, Peter Graf

**Affiliations:** 1 Department of Psychiatry, University of British Columbia, Vancouver, British Columbia, Canada; 2 Department of Psychology, University of British Columbia, Vancouver, British Columbia, Canada; University of Granada, Spain

## Abstract

While the effects of cannabis use on retrospective memory have been extensively examined, only a limited number of studies have focused on the links between cannabis use and prospective memory. We conducted two studies to examine the links between cannabis use and both time-based and event-based prospective memory as well as potential mechanisms underlying these links. For the first study, 805 students completed an online survey designed to assess cannabis consumption, problems with cannabis use indicative of a disorder, and frequency of experiencing prospective memory failures. The results showed small to moderate sized correlations between cannabis consumption, problems with cannabis use, and prospective memory. However, a series of mediation analyses revealed that correlations between problems with cannabis use and prospective memory were driven by self-reported problems with retrospective memory. For the second study, 48 non-users (who had never used cannabis), 48 experimenters (who had used cannabis five or fewer times in their lives), and 48 chronic users (who had used cannabis at least three times a week for one year) were administered three objective prospective memory tests and three self-report measures of prospective memory. The results revealed no objective deficits in prospective memory associated with chronic cannabis use. In contrast, chronic cannabis users reported experiencing more internally-cued prospective memory failures. Subsequent analyses revealed that this effect was driven by self-reported problems with retrospective memory as well as by use of alcohol and other drugs. Although our samples were not fully characterized with respect to variables such as neurological disorders and family history of substance use disorders, leaving open the possibility that these variables may play a role in the detected relationships, the present findings indicate that cannabis use has a modest effect on self-reported problems with prospective memory, with a primary problem with retrospective memory appearing to underlie this relationship.

## Introduction

Cannabis is the most widely used illicit substance in the world, with an estimated 129 to 191 million users worldwide [1]. Both conventional wisdom and empirical research suggest that cannabis use negatively impacts cognitive functioning. Perhaps the most well-described effects of cannabis consumption are with regard to executive functioning and retrospective memory. A recent review has indicated that long-term, heavy, or chronic cannabis users display impairments in encoding, storage, manipulation, and retrieval, and moreover, these impairments are strikingly similar to those associated with acute cannabis intoxication [2].

Studies examining the impact of cannabis use on memory functioning have largely been restricted to the domain of retrospective memory–the ability to remember previously learned information, facts, and events. As a result, little is understood about the influence of cannabis use on prospective memoryE−the ability to formulate, retain, recollect, and carry out future plans and intentions at the appropriate time or in the appropriate context [3], [4]. Prospective memory tasks pervade our everyday lives impacting our occupational, social, and personal functioning. In light of the widespread use of cannabis and the importance of prospective memory for everyday functioning, the primary goal of this paper was to further examine the links between cannabis use and prospective memory, as well as the mechanisms that may underlie these links.

Research examining self-reported problems with prospective memory in cannabis users has produced equivocal results, with some studies demonstrating significant correlations between cannabis use and self-reported problems with prospective memory [5], [6], [7] and other studies failing to reveal such effects [8], [9]. Though intriguing, the primary focus of most of the existing studies has been to examine the influence of ecstasy use on prospective memory [5], [6], [8], rather than cannabis use specifically. As such, some of the reported results may have been biased by the use of non-representative groups of poly-drug cannabis users.

Cannabis users' performance on objective time- and event-based prospective memory tests has been assessed in three previous studies. As their names imply, time-based tests are those that require execution at a specific time, while event-based tests are those that require execution upon the occurrence of a specific event [10], [11]. McHale and Hunt (2008) reported that cannabis users showed significantly more failures than non-users on a long-term time-based prospective memory test and that their performance on a short-term time-based prospective memory test was significantly more delayed. In contrast, no significant effects were discovered on an event-based prospective memory test, although a trend toward such an effect was reported [12]. Similarly, Bartholomew et al. (2010) found that cannabis-only users recalled significantly fewer location-action combinations than non-users on a video-based prospective memory test [9]. In contrast, Hadjiefthyvoulou et al. (2011) compared the performance of cannabis-only users, ecstasy/poly-drug users, and non-drug users on the Cambridge Prospective Memory Test (CAMPROMPT) and found no significant differences between the cannabis-only users and non-drug users on either the time- or event-based tests [13].

Together, these findings suggest that cannabis use may negatively impact prospective memory. In the brain, the psychoactive constituents of cannabis (notably Δ^9^-tetrahydrocannabinol) exert their effects via activation of cannabinoid CB_1_ receptors, which are inhibitory G-protein-coupled receptors that are widely expressed in brain structures known to be implicated in prospective memory, such as the hippocampus and prefrontal cortex [14]. Given the established role of CB_1_ receptors in the acquisition, encoding, and retrieval of memories [15], the detected effects of cannabis use on prospective memory may be mediated by changes in this receptor population.

Alternatively, it is possible that the detected effects may reflect a selection bias, or extraneous variables associated with cannabis use that cannot be controlled for using random assignment. For instance, use of other drugs could obscure the direct influence of chronic cannabis use on prospective memory. Among cannabis users, the prevalence of alcohol and other drug use is particularly high, and furthermore, alcohol, ecstasy and poly-drug use have been shown to negatively impact prospective memory [6], [13], [16]–[19]. The presence of negative mood states could also mediate the link between cannabis use and prospective memory. For instance, chronic heavy cannabis use has been associated with an increased risk of depressive-like symptoms [20], and moreover, anxiety and depressed mood are associated with problems with prospective memory [21]–[24].

The impact of cannabis use on retrospective memory must also be considered. Prospective memory involves two distinct components, a prospective component that involves remembering that a task needs to be performed at the appropriate moment, and a retrospective component that involves remembering what task needs to be performed [3], [11]. The experience of remembering to stop at the grocery store only to forget what you intended to purchase is an example of the success of the prospective component and failure of the retrospective component. Given the dual demands implicated in successful prospective memory performance, it is possible that the previously reported deficits in chronic cannabis users' prospective memory are secondary to established problems in retrospective memory. Indeed, two of the previous investigations that examined objective prospective memory test performance in cannabis users relied on tests that place a greater demand on the retrospective component, rather than on the prospective component [9], [13]. For instance, the prospective memory test used by Bartholomew et al. (2010) required participants to learn 17 location-action associations and to record the relevant location-action pair upon presentation of each location in a 10-minute video [9]. The reported deficits in cannabis users on this test may reflect a failure of associative learning ability in cannabis users, rather than a deficit in prospective memory per se. Indeed, performance on this test does not correlate with more traditional tests of prospective memory [25].

We set out to increase understanding of the links between cannabis use and prospective memory in two independent studies. The objective of the first study was to examine the relationship between cannabis use and self-reported problems with time-and event-based prospective memory. While previous researchers have examined the link between cannabis use and self-reported problems with prospective memory, they have exclusively done so using the Prospective Memory Questionnaire (PMQ) [26], an instrument which focuses on event-based prospective memory. In light of McHale and Hunt's (2008) findings of deficits on time- but not event-based tests, we included a self-report inventory designed to assess time-based prospective memory [22]. We also included both the PMQ and the Prospective and Retrospective Memory Questionnaire (PRMQ) [27] and utilized the retrospective memory subscale of the PRMQ to examine the mediating effects of self-reported problems with retrospective memory.

The objective of the second study was to investigate whether cannabis users exhibit deficits on objective event- and time-based prospective memory tests, which place relatively low demands on the retrospective component of prospective memory. In mind of previous findings showing an association between cannabis use and delayed responses on a time-based prospective memory test [10], we also measured the degree to which responses on both a time- and event-based prospective memory test were delayed. Finally, we examined possible mediating factors that could account for cannabis-related prospective memory deficits, including problems with retrospective memory, alcohol and other drug use, and the presence of symptoms of anxiety and depression.

## Materials and Methods

### Study One

#### Ethics Statement

We conducted this online survey with the approval of the University of British Columbia (UBC) Behavioural Ethical Review Board. Participants were recruited from the Department of Psychology human subject pool and received course credit in return for their participation. All participants provided electronic informed consent. The consent form was shown on the first page of the online survey followed by the statement ‘I agree to participate’ and a button labelled ‘Yes’. The remainder of the survey appeared only after participants clicked to indicate their consent to participate. To ensure that participants remained anonymous, no identifying information was solicited in the survey.

#### Participants

A total of 805 undergraduate students from UBC completed the online survey. Respondents ranged from 17 to 39 years of age with a mean of 20.44 years (*SD* = 2.34). The number of years of postsecondary education participants had completed ranged from 1 to 10, with a mean of 2.87 years (*SD* = 1.37). There were 291 male (36.10%) and 513 female (63.70%) participants. One participant failed to identify his/her gender.

#### Cannabis Use Items

We included several items at the beginning of the survey to characterize participants' current and lifetime use of cannabis. These items were used to assess whether individuals had ever used cannabis, whether they were under the influence of cannabis when completing the survey, as well as their average quantity and frequency of cannabis use. Quantity of cannabis use was measured with two items. The first asked: “On average, how much marijuana do you smoke at a time? Note: if you use marijuana in forms other than a joint indicate the quantity that is roughly equivalent”. Response options ranged from 0, indicating “I never use marijuana” to 12, indicating “More than 4 joints”. The second item stated: “On average how many grams of marijuana do you smoke in one month? Note: there are 28 grams in an ounce”. Response options ranged from 0, indicating “I never use marijuana” to 12, indicating “More than 2 ounces”. Frequency of cannabis use was also measured with two items. The first read: “On average how frequently do you use marijuana?” Response options ranged from 0, indicating “Never” to 11, indicating “More than 5 times a day”. Finally participants were ask to respond to the question: “Which of the following best characterizes your use of marijuana?” using a scale ranging from 0, indicating “I never use marijuana” to 3, indicating “I use marijuana frequently”. The first principal component of these four items was computed as an index of cannabis consumption. All four items showed factor loadings greater than.90.

#### Marijuana Screening Inventory (MSI X) Revised

The MSI X is a 31-item screening inventory designed to assess maladaptive problems associated with cannabis use specifically (e.g., problems with relationships, problems with the law resulting from the use of cannabis) [28]. Since the inventory measures problems associated with the use of cannabis, those individuals who reported that they have never used cannabis were not administered the inventory.

#### Prospective Memory Questionnaire (PMQ)

The PMQ is a valid and reliable 52-item self-report inventory for assessing prospective memory in everyday life [26]. The questionnaire contains four subscales that measure the frequency with which individuals experience failures on long-term episodic prospective memory tasks (i.e., tasks that need to be performed once after a long delay), short-term habitual tasks (i.e., tasks that need to be performed routinely after a short delay), internally-cued tasks (i.e., tasks with no salient external cue for prompting retrieval), and the frequency individuals use prospective memory aiding strategies (e.g., reminder notes). We used a slightly simplified 6-point rating scale as participants in previous studies have reported difficulties using the original scales. This simplified version has higher reported test-retest reliabilities than the original version [20], [24].

#### Prospective and Retrospective Memory Questionnaire (PRMQ)

The PRMQ is a 16-item valid and reliable self-report inventory designed to assess various memory failures [27], [29]. The questionnaire contains two subscales, one focusing on prospective memory and the other on retrospective memory.

#### Time-Cued Prospective Memory Questionnaire (TCPMQ)

The TCPMQ is a reliable 89-item inventory designed to assess various aspects of time-based prospective memory [22]. The inventory has three sections. For the first section participants rate how frequently they experience various time-based prospective memory failures. The second section assesses punctuality. Participants are presented with the same time-based prospective memory task descriptions contained in the first section and are asked to rate how punctual they usually are when they perform the activity. The third section assesses participants' use of time-based prospective memory aiding strategies (e.g., alarms).

#### Psychopathic Personality Inventory (PPI)

Ten items from the Deviant Responding validity subscale of the PPI [30] were randomly interspersed throughout the survey. These items are not indicative of psychopathy, rather they are bizarre items (e.g., “When I am under stress, I often see large, red, rectangular shapes moving in front of my eyes”) used to detect individuals who are carelessly or randomly responding to the survey questions. Participants who made more than three unusual endorsements were deemed random responders and their data were discarded.

### Study Two

#### Ethics Statement

We conducted the second study with the approval of the UBC Behavioural Ethical Review Board. All participants provided written informed consent and participants received course credit in return for their participation.

#### Participants and Design

For the second study, we used a cross sectional design. Undergraduate students who had not participated in the first study were recruited from the Department of Psychology human subject pool by means of three separate advertisements. One called for individuals who had never used cannabis (whom we will refer to as ‘non-users’), another called for individuals who had tried cannabis five times or fewer over the course of their lives (whom we will refer to as ‘experimenters’), and the final advertisement sought individuals who used cannabis three or more times a week for at least one year (whom we will refer to as ‘chronic users’). The group of experimenters was included in an effort to control for potential differences between chronic cannabis users and non-users. Presumably, the experimenters had not used cannabis enough for it to cause any cognitive impairment, however, since they had all at least experimented with the drug, the inclusion of this group allowed us to control for some of the factors related to the decision to try cannabis. Participants' suitability for placement within these three categories was confirmed using their survey responses and those participants who did not meet these criteria were excluded. We also excluded participants who were identified as random responders using the PPI and four participants who indicated that they were under the influence of cannabis. No other exclusion criteria were applied. By this process we recruited a total of 48 non-users, 48 experimenters, and 48 chronic users.

Participants' demographic characteristics were comparable to those reported in study one. Participants ranged from 17 to 33 years of age with an overall mean of 20.31 years (*SD* = 2.62). The number of years of postsecondary education participants had completed ranged from 1 to 7 years with an overall mean of 2.31 years (*SD* = 1.21). Verbal IQ was estimated using the North American Adult Reading Test (NAART) [31] and the following equation, 128.7 − .89 x NAART Errors [32]. Estimated verbal IQ scores ranged from 83 to 122 with an overall mean of 102.83 (*SD* = 8.69). There were 54 male (36.49%) and 94 female (63.51%) participants. As shown in Table 1, a comparison of demographic characteristics across groups revealed no significant differences in age. However, groups did differ with respect to years of postsecondary education completed, estimated verbal IQ, gender, and English as second language status (ESL status). Follow-up analyses revealed that the experimenters had completed significantly more years of education than the non-users and chronic users, who did not differ significantly. The chronic users contained significantly more native English speakers than the non-users and experimenters, who did not differ significantly. Finally, the non-users had significantly lower estimated verbal IQ scores and contained fewer men than the experimenters and chronic users, who did not differ significantly. Due to these differences the influence of these demographic variables on our effects of interest was examined.

**Table 1:** Demographic characteristics of non-users, experimenters, and chronic users.

	Non-Users (N = 48)	Experimenters (N = 48)	Chronic Users (N = 48)	
Age	19.71 (2.59)	20.75 (2.78)	20.42 (2.52)	*F* (2,141) = 1.96, p = .14
Years of University	1.94 (1.24)^a^	2.71 (1.22)^b^	2.22 (1.06)^a^	***F*** ** (2,141) = 5.25, p = .006**
Estimated Verbal IQ	99.00 (9.13)^a^	103.63 (8.16)^b^	105.86 (7.41)^b^	***F*** ** (2,141) = 8.61, p<.001**
% Female	79.17%^a^	60.42%^b^	47.92%^b^	**χ^2^ (2) = 8.01, p = .02**
% English First Language	45.83%^a^	70.83%^a^	81.25%^b^	**χ^2^ (2) = 14.17, p = .001**

Note: bold indicates *p*≤05. Verbal IQ was estimated using the NAART and the following equation, 128.7−.89 x NAART Errors. Groups with different subscripts differed significantly.

Using MSI X scores, 43 (89.58%) of the chronic users were found to be at high risk for cannabis abuse and/or dependence, 4 (8.33%) were found to be at moderate risk and 1 (2.08%) was found to be at low risk. Fourteen (29.17%) of the chronic users reported using cannabis 3 times a week, 20 (41.67%) reported using it once a day, and 14 (29.17%) reported using it more than once a day.

Participants were tested individually. Each completed a paper-and-pencil version of the survey that was used in study one and the NAART [31]. In addition, they completed the following questionnaires, retrospective memory tests, and prospective memory tests.

#### Questionnaires

The Michigan Alcohol Screening Test [33] was used to assess lifetime problems with alcohol and alcoholism. The Psychoactive Drug History Questionnaire [34] was used to assess the overall frequency of use of drugs other than cannabis in the past 6 months. The Beck Depression Inventory [35], [36] was used to assess symptoms of depression and the State Trait Anxiety Inventory [37] was used to assess symptoms of state and trait anxiety.

#### Retrospective Memory Tests

The Digit Span Backwards Test (DSB) was used to assess working memory [38] and total scores on the first three trials of the Rey Auditory Verbal Learning Test (AVLT) were used to assess explicit episodic retrospective memory [39]. To avoid potential ceiling effects five additional items from an alternate version of the AVLT were added to the standard 15 item list. We also used a picture recognition test [40]. For this test participants were shown 120 pictures of objects. After a delay of one hour, they were given a recognition test containing 120 objects from the original list and 124 new objects. The proportion of correct identifications was scored.

#### Prospective Memory Tests

To assess event-based prospective memory we used the Fruit Prospective Memory Test [40]. Participants were informed that sometime during the course of the experiment they would see pictures of fruit. They were instructed to stop whatever they were doing and press the ‘p’ key on the computer keyboard whenever they encounter such a picture. There were four pictures of fruit embedded within the recognition portion of the picture recognition test, and participants were given one point for each successful trial.

As a more ecologically valid event-based prospective memory test, we also used a Reminder Prospective Memory Test [22]. For this test participants were asked to give the experimenter a reminder as soon as the cognitive tests were completed. Following this request they were given a description of the last cognitive test that they would complete. In an attempt to manipulate motivation to complete the test, we employed two versions; a high motivation and a low motivation version. In the high motivation version participants were asked to remind the experimenter to submit their research participation credit. In the low motivation version they were asked to remind the experimenter to send an email to her supervisor. Participants who provided a timely reminder were given a score of 2 on the test, those who provided a reminder at the wrong time were given a score of 1, and those who completely failed to give the experimenter the reminder were given a 0 on the test. The number of minutes early/late the reminder was given was also recorded.

Finally, as a measure of time-based prospective memory we used a Call In Prospective Memory Test [40]–[42]. For this test participants were required to call the lab exactly one week after the in-lab testing session during a one hour time window selected by the participant. Those participants who successfully called on the correct day and time were given a score of 2, those who called at the incorrect time were given a score of 1, and those who completely failed to call were given a score of 0. For those who called at the incorrect time, the number of minutes early/late they called was also recorded.

## Results

### Study One

#### Data Preparation

Forty of the 805 respondents (4.97%) from study one indicated that they were under the influence of cannabis while completing the online survey and 61 (7.58%) were deemed random responders. These participants were excluded from all subsequent analyses. Nine of the participants who indicated that they were under the influence of cannabis were random responders so a total of 92 participants were excluded.

Of the remaining 713 participants, 160 (22.44%) received a score of 6 or higher on the MSI X indicating a high risk for cannabis abuse and/or dependence. Ninety-six (13.46%) received a score of 3–5 indicating a moderate risk. Eighty-six (12.06%) received a score of 1–2 indicating a low risk and 34 (4.77%) received a score of 0 indicating no risk [28]. Finally 337 participants (47.27%) did not complete the MSI X inventory.

Due to the inclusion of a “not applicable” response option on many of the questionnaires and the presence of other sporadic missing data, averages rather than sums were used to derive the subscales of the various prospective memory questionnaires. Participants with too little data to compute a meaningful subscale score were excluded from the relevant analyses. Each of the questionnaire subscale scores were examined for univariate outliers. Fewer than 1% of the data were outliers, nevertheless, outliers were replaced with the nearest non-outlying value, specifically a score either −3 or +3 standard deviations away from the corresponding mean. The pattern of results was not affected by this adjustment to outliers. While the numerous correlations between the various prospective memory subscales makes a full Bonferroni correction inappropriate [43], the more conservative alpha level of .01 was used to control for inflation of Type I error.

#### Correlations Between Cannabis Use and Prospective Memory Failures

A series of correlation analyses were conducted to assess the relationships between cannabis consumption, problems with cannabis use, and self-reported prospective memory failures. As shown in Table 2, small but significant correlations were detected between cannabis consumption and the episodic prospective memory subscale of the PMQ, the internally-cued prospective memory subscale of the PMQ, the prospective memory subscale of the PRMQ, and the punctuality subscale of the PRMQ. When the analyses were restricted to only those who reported using cannabis, none of the correlations remained significant. In contrast, scores on the MSI X, which was only administered to individuals who reported using cannabis, showed significant correlations with all of the questionnaire subscales except for those measuring the use of memory aiding strategies.

**Table 2:** Correlations between cannabis consumption, problems with cannabis use, and self-reported problems with prospective memory.

	Full Sample (N = 713)	Cannabis Users (N = 376)
Questionnaire and Subscale	Cannabis Consumption	Problems with Use
**PMQ**		
Episodic Prospective Memory	***r*** ** (711) = .15, ** ***p*** **<.001, ** ***r^2^*** ** = .02**	***r*** ** (374) = .29, ** ***p*** **<.001, ** ***r^2^*** ** = .08**
Habitual Prospective Memory	*r* (709) = .07, *p* = .06, *r^2^* = .005	***r*** ** (373) = .29, ** ***p*** **<.001, ** ***r^2^*** ** = .08**
Internally-Cued Prospective Memory	***r*** ** (709) = .12, ** ***p*** ** = .001, ** ***r^2^*** ** = .01**	***r*** ** (373) = .34, ** ***p*** **<.001, ** ***r^2^*** ** = 12**
Memory Aiding Strategies	*r* (709) = .00, *p* = .94, *r^2^* = .00	*r* (373) = .06, *p* = .28, *r^2^* = .003
**PRMQ**		
Prospective Memory	***r*** ** (704) = .11, ** ***p*** ** = .003, ** ***r^2^*** ** = .01**	***r*** ** (371) = .30, ** ***p*** **<.001, ** ***r^2^*** ** = .09**
**TCPMQ**		
Time-Based Prospective Memory	*r* (710) = .04, *p* = .30, *r^2^* = .002	***r*** ** (373) = .19, ** ***p*** **<.001, ** ***r^2^*** ** = .04**
Punctuality	***r*** ** (710) = .18, ** ***p*** **<.001, ** ***r^2^*** ** = .03**	***r*** ** (374) = .28, ** ***p*** **<.001, ** ***r^2^*** ** = .08**
Memory Aiding Strategies	*r* (710) = −.02, *p* = .61, *r^2^* = .0004	*r* (374) = −.02, *p* = .69, *r^2^* = .0004

Note: bold indicates *p*≤.01. PMQ = Prospective Memory Questionnaire; PRMQ = Prospective and Retrospective Memory Questionnaire; TCPMQ = Time−Cued Prospective Memory Questionnaire. Higher scores on the prospective memory questionnaires indicate more frequent prospective memory failures or greater use of memory aiding strategies. Higher scores on the punctuality subscale of the TCPMQ indicate performance of tasks later. Higher scores on the cannabis consumption and problems with use variables indicate greater cannabis consumption and more problems associated with cannabis use, respectively.

#### Mediation Analyses

To determine whether problems with retrospective memory mediated any of the correlations reported above we first conducted two separate regression analyses. The results showed that problems with cannabis use was a significant predictor of self-reported problems with retrospective memory, β = .33, *p*<.001, *r^2^* = 11. In contrast, the predictive power of cannabis consumption on problems with retrospective memory fell just shy of our conservative alpha level, β = .09, *p* = .02, *r^2^* = 01, and thus was ruled out as a potential mediator.

Sobel tests of mediation [44], which are based on Baron and Kenny's [45] method of detecting mediators, showed that self-reported problems with retrospective memory mediated the relationship between problems with cannabis use and episodic prospective memory, *z* = 5.78, *p*<.001, habitual prospective memory, *z* = 5.34, *p* = .002, internally-cued prospective memory, *z* = 6.04, *p*<.001, the prospective memory subscale of the PRMQ, *z* = 6.11, *p*<.001, time-based prospective memory, *z* = 5.51, *p*<.001, and punctuality, *z* = 3.66, *p*<.001.

### Study Two

#### Data Preparation

Once again, due to inclusion of a “not applicable” response option on several of the questionnaires and the presence of other sporadic missing data, averages rather than sums were used to derive the subscales of most of the various inventories. Participants with too little data to compute a meaningful subscale score were excluded from the relevant analyses. Fewer than 1% of the data represented univariate outliers, nevertheless, outliers were replaced with the nearest non-outlying value, specifically a score either −3 or +3 standard deviations away from the corresponding mean. The pattern of results was not affected by this adjustment to outliers. An alpha level of .01 was used to control for inflation in Type I error.

#### Correlations Between Cannabis Use and Prospective Memory Failures

In an attempt to replicate and extend our findings from the first study, we examined the correlations between cannabis consumption, problems with cannabis use, and both self-reported prospective memory failures and objective prospective memory test performance. The results in Table 3 show that the cannabis consumption variable was significantly correlated with self-reported problems with internally-cued prospective memory. Consistent with study one, analyses restricted to only the chronic users failed to reveal any significant correlations between cannabis consumption and prospective memory. Also in the chronic users group MSI X total scores were significantly correlated with the episodic prospective memory and internally-cued prospective memory subscales of the PMQ, the prospective memory subscale of the PRMQ, and the time-based prospective memory and punctuality subscales of the TCPMQ.

**Table 3:** Results of correlations between cannabis consumption, problems with cannabis use, and prospective memory.

	Full Sample (N = 144)	Chronic Users (N = 48)
	Cannabis Consumption	Problems with Use
**PMQ**		
Episodic Prospective Memory	*r* (142) = .20, *p* = .02, *r^2^* = .04	***r*** ** (46) = .49, ** ***p*** **<.001, ** ***r^2^*** ** = .24**
Habitual Prospective Memory	*r* (142) = .07, *p* = .39, *r^2^* = .005	*r* (46) = .24, *p* = .09, *r^2^* = .06
Internally-Cued Prospective Memory	***r*** ** (142) = .23, ** ***p*** ** = .006, ** ***r^2^*** ** = .05**	***r*** ** (46) = .45, ** ***p*** ** = .001, ** ***r^2^*** ** = .20**
Memory Aiding Strategies	*r* (142) = .05, *p* = .53, *r^2^* = .003	*r* (46) = .12, *p* = .40, *r^2^* = .01
**PRMQ**		
Prospective Memory	*r* (142) = .19, *p* = .02, *r^2^* = .04	***r*** ** (46) = .38, ** ***p*** ** = .008, ** ***r^2^*** ** = .14**
**TCPMQ**		
Time-Based Prospective Memory	*r* (142) = .01, *p* = .93, *r^2^* = .0001	***r*** ** (46) = .40, ** ***p*** ** = .005, ** ***r^2^*** ** = .16**
Punctuality	*r* (142) = .15, *p* = .07, *r^2^* = .02	***r*** ** (46) = .47, ** ***p*** ** = .001, ** ***r^2^*** ** = .22**
Memory Aiding Strategies	*r* (142) = .12, *p* = .14, *r^2^* = .01	*r* (46) = .01, *p* = .93, *r^2^* = .0001
**Prospective Memory Tests**		
Reminder Test	*r* (142) = .07, *p* = .41, *r^2^* = .005	*r* (46) = .19, *p* = .19, *r^2^* = .04
Call In Test	*r* (142) = –.06, *p* = .50, *r^2^* = .004	*r* (46) = –.31, *p* = .03, *r^2^* = .10
Fruit Test	*r* (142) = .04, *p* = .65, *r^2^* = .002	*r* (46) = .00, *p* = .98, *r^2^* = .00

Note: bold indicates p≤.01. PMQ = Prospective Memory Questionnaire; PRMQ = Prospective and Retrospective Memory Questionnaire; TCPMQ = Time−Cued Prospective Memory Questionnaire. Higher scores on the prospective memory questionnaires indicate more prospective memory failures or greater use of memory aiding strategies. Higher scores on the cannabis consumption and problems with use variables indicate greater cannabis consumption and more problems associated with cannabis use, respectively. Higher scores on the prospective memory tests indicate better prospective memory test performance.

We also examined whether group differences in estimated verbal IQ, gender, years of postsecondary education or ESL status were responsible for these effects by correlating each of these variables with ratings on the episodic prospective memory and internally-cued prospective memory subscales of the PMQ, the prospective memory subscale of the PRMQ, and the time-based prospective memory and punctuality subscales of the TCPMQ. None of the correlations were significant, and as such group differences in estimated verbal IQ, gender, years of postsecondary education and ESL status cannot be responsible for any of the correlations reported above [45].

#### Mediation Analyses

Eight separate regression analyses using either cannabis consumption or problems with cannabis use to predict performance on the AVLT, PRT, DSB and PRMQ retrospective memory subscale showed that cannabis consumption and problems with cannabis use were both significant predictors of AVLT performance, β = −.27, *p* = .001, *r^2^* = .07; β = −.43, *p* = .002, *r^2^* = .18, respectively. Problems with cannabis use was also a significant predictor of self-reported problems with retrospective memory, β = .40, *p* = .005, *r^2^* = .16. No other effects were significant at the .01 level. Follow-up Sobel tests showed that performance on the AVLT was not a significant mediator of any of the correlations between cannabis consumption, problems with cannabis use, and prospective memory. However, consistent with the findings from the first study, self-reported problems with retrospective memory was a significant mediator of the correlations between problems with cannabis use and episodic prospective memory, *z* = 2.78, *p* = .005, internally-cued prospective memory, *z* = 2.85, *p* = .004, the prospective memory subscale of the PRMQ, *z* = 2.82, *p* = .004, time-based prospective memory, *z* = 2.57, *p* = .01, and punctuality, *z* = 2.48, *p* = .01.

A similar set of analyses was conducted to examine whether depression, state anxiety, trait anxiety, problems with alcohol, or use of other drugs could account for the correlations between cannabis use and prospective memory. The analyses revealed that cannabis consumption was only a significant predictor of problems with alcohol, β = .46, *p*<.001, *r^2^* = .21, and use of other drugs, β = .54, *p*<.001, *r^2^* = .29, and moreover that the correlations between cannabis consumption and self-reported problems with internally-cued prospective memory was mediated by these variables, *z* = 2.80, *p* = .005; *z* = 2.65, *p* = .008, respectively. In contrast, none of the examined variables significantly mediated the correlations between problems with cannabis and prospective memory.

#### Group Differences in Objective Prospective Memory Test Performance

A preliminary analysis of the Reminder Prospective Memory Test showed no interaction between group and test type (high motivation version, low motivation version), *χ^2^* (2) = .06, *p* = .97, so the two test types were combined. As shown in Table 4, comparisons of the three groups' prospective memory test performance indicated no significant effect of group on any of the prospective memory tests. While a less conservative alpha would have revealed a significant effect on the Fruit Prospective Memory Test, follow-up exploratory posthoc analyses showed that the chronic users performed similarly to the non-users and experimenters. The effect was driven by the fact that the non-users performed worse than the experimenters. The non-parametric Kruskal-Wallis h test revealed the same pattern of results on this test. Due to a low frequency of late reminders (all but one of the off-time reminders made by the chronic users were early rather than late), data pertaining to delay in responses could not be meaningfully analyzed.

**Table 4:** Results of comparisons of non-users', experimenters', and chronic users' prospective memory.

		Non-Users (N = 48)	Experimenters (N = 48)	Chronic Users (N = 48)	
**Prospective Memory Tests**					
Reminder Test	On-Time	62.50%	64.58%	68.75%	*χ^2^* (4) = 2.80, *p* = .59, *φ_c_* = .10
	Off-Time	14.58%	20.83%	10.42%	
	Failure	22.92%	14.58%	20.83%	
Call In Test	On-Time	37.50%	43.75%	33.33%	*χ^2^* (4) = 1.18, *p* = .88, *φ_c_* = .06
	Off-Time	8.33%	8.33%	8.33%	
	Failure	54.17%	47.92%	58.33%	
Fruit Test		1.08 (1.50)	1.85 (1.69)	1.23 (1.55)	*F* (2,140) = 3.20, *p = *.04, η^2^ = .04
**PMQ**					
Episodic Prospective Memory		2.29 (.54)	2.45 (.65)	2.61 (.60)	*F* (2, 141) = 3.57, *p* = .03, *η^2^* = .05
Habitual Prospective Memory		1.33 (.33)	1.35 (.37)	1.41 (.39)	*F* (2, 141) = .58, *p* = .56, *η^2^* = .008
Internally-Cued Prospective Memory		2.13 (.64)^a^	2.06 (.56)^a^	2.46 (.82)^b^	***F*** ** (2, 141) = 4.63, ** ***p*** ** = .01, ** ***η^2^*** ** = .06**
Memory Aiding Strategies		2.85 (.74)	2.73 (.80)	2.96 (.87)	*F* (2, 141) = 3.91, *p* = .40, *η^2^* = .01
**PRMQ**					
Prospective Memory		2.44 (.60)	2.48 (.60)	2.73 (.73)	*F* (2, 141) = 1.16, *p* = .07, *η^2^* = .04
**TCPMQ**					
Time-Based Prospective Memory		1.66 (.41)	1.69 (.43)	1.68 (.37)	*F* (2, 141) = .05, *p* = .95, *η^2^* = .001
Punctuality		2.76 (.38)	2.87 (.45)	2.94 (.31)	*F* (2, 141) = 2.61, *p* = .08, *η^2^* = .04
Memory Aiding Strategies		2.44 (.61)	2.60 (.84)	2.69 (.64)	*F* (2, 141) = 1.54, *p* = .22, *η^2^* = .02

Note: bold indicates p≤.01. Groups with different subscripts differed significantly. PMQ = Prospective Memory Questionnaire; PRMQ = Prospective and Retrospective Memory Questionnaire; TCPMQ = Time−Cued Prospective Memory Questionnaire. Higher scores on the prospective memory questionnaires indicate more frequent prospective memory failures or greater use of memory aiding strategies. Scores of 3 on the TCPMQ punctuality subscale reflect on-time performance, scores below 3 indicate early performance and scores above 3 indicate late performance. Higher scores on the Fruit Prospective Memory Test indicate better performance.

#### Group Differences in Self-Reported Prospective Memory Failures

As shown in Table 4, a series of one-way ANOVAs comparing the three groups' ratings of self-reported failures of prospective memory revealed a significant effect only on the internally-cued subscale of the PMQ. Follow-up comparisons indicated that chronic users reported significantly more problems than both non-users and experimenters, whose ratings did not differ. ANCOVA analyses re-examining these effects after controlling for group differences in gender, years of post-secondary education, and verbal IQ slightly enhanced the differences across the groups, *F* (2, 137)  = 6.12, *p* = .003, *η^2^* = .08.

#### Accounting for Group Differences in Self-Reported Prospective Memory Failures

To determine whether the group differences on the internally-cued prospective memory subscale of the PMQ could be explained by group differences in retrospective memory, we conducted a series of ANOVAs to examine whether the groups differed on the various measures of retrospective memory (AVLT, PRT, DSB or PRMQ retrospective memory subscale). Groups were found to differ significantly only with respect to self-reported problems with retrospective memory, *F* (1, 141)  = 3.91, *p* = .02, *η^2^* = .05, and performance on the AVLT, *F* (2,141)  = 7.12, *p* = .001, *η^2^* = .09. An ANCOVA examining group differences on the internally-cued prospective memory subscale, after controlling for self-reported problems with retrospective memory and deficits on the AVLT, showed no effect across the groups, *F* (2, 139) = 1.30, *p* = .27, *η^2^* = .02.

Finally, we conducted a series of ANOVAs to examine the influence of problems with alcohol, use of other drugs, depression, state anxiety, and trait anxiety on self-reported problems with internally-cued prospective memory. Groups were found to differ significantly only with respect to drinking problems, *F* (2, 141)  = 26.78, *p*<.001, *η^2^* = .23, and use of other drugs, *F* (2, 141)  = 31.09, *p* <.001, *η^2^* = .31. An ANCOVA examining group differences on internally-cued prospective memory, after controlling for problems with drinking and use of other drugs, showed no significant effect, *F* (2, 139)  = 1.03, *p*  = .36, *η^2^* = .01.

## Discussion

While the effects of cannabis use on retrospective memory have been extensively examined, only a small number of studies have investigated the links between cannabis use and prospective memory. In light of the pervasive use of cannabis and the importance of prospective memory in our everyday life functioning, the present two studies were conducted to further our understanding of the relationships between cannabis use and prospective memory.

In the first study we examined the links between cannabis use and self-reported problems with event- and time-based prospective memory, as well as the extent to which these relationships are mediated by retrospective memory. The results revealed significant correlations between cannabis consumption and self-reported failures on the episodic and internally-cued subscales of the PMQ, the prospective memory subscale of the PRMQ, and the punctuality subscale of the TCPMQ. However the correlations were small, indicating that cannabis consumption accounts for only 1–3% of the variance in self-reported problems with these aspects of prospective memory. Moreover, when individuals who reported never using cannabis were excluded from the analyses these correlations were no longer significant. This indicates that the presence versus absence of cannabis consumption may be driving these relationships more than the amount of cannabis consumed per se, and possibly that some variable associated with the choice to use cannabis is influencing the correlations. In contrast, MSI X total scores – which quantify problems with cannabis use indicative of a cannabis abuse or dependence disorder – showed consistent moderate sized correlations with all of the various subscales measuring the frequency of experiencing prospective memory failures, suggesting that problems with cannabis use accounts for 4–12% of the variance in everyday life prospective memory failures. Nevertheless, the results of subsequent mediation analyses demonstrated that each of the correlations with the MSI X was mediated by self-reported problems with retrospective memory.

The results of the correlation analyses from the second study were largely consistent with those from the first study. Cannabis consumption was significantly correlated with self-reported failures on the internally-cued subscale of the PMQ, accounting for 5% of the variance. However, this effect was found to be mediated by use of alcohol and other drugs. Due to the reduction in power resulting from our smaller sample size in this study, the correlations between cannabis consumption and the episodic prospective memory subscale of the PMQ, the prospective memory subscale of the PRMQ, and the punctuality subscale of the TCPMQ failed to reach significance at our conservative. 01 alpha level. Nevertheless, the magnitude of these correlations and the corresponding effect sizes were, for the most part, similar to or slightly higher than those revealed in the first study. Also consistent with the first study, problems with cannabis use showed larger and more consistent correlations with the various self-report measures of prospective memory, and self-reported problems with retrospective memory were once again found to mediate each of the correlations.

It is intriguing that in both studies problems with cannabis use showed larger and more consistent correlations with the various self-report measures of prospective memory than cannabis consumption. It is particularly interesting given that this measure was only administered to individuals who reported using cannabis and when analyses pertaining to cannabis consumption were restricted to this same group the correlations with cannabis consumption were reduced to non-significance. It may be that some individuals are able to use relatively large amounts of cannabis frequently without it creating the various problems associated with an abuse or dependence disorder and without it adversely impacting their prospective memory functioning in everyday life. However, this is largely speculative and it is also possible that our measure of cannabis consumption was simply inferior to the more structured MSI X used to measure problems with cannabis use. Regardless, researchers would be wise to consider using this measure of problems with cannabis use in future investigations.

The second study also featured a cross-sectional design, in which the objective prospective memory test performance and subjective ratings of non-users, experimenters, and chronic users of cannabis were compared. Groups showed no significant differences in their performance on any of the event- or time-based prospective memory tests. In contrast to the findings of McHale and Hunt (2008) our objective prospective memory tests also failed to reveal any evidence of delayed performance in the chronic users group. Indeed, all but one of the off-time responses in the chronic users group reflected early rather than late performance. With respect to self-reported problems with prospective memory, only the internally-cued subscale of the PMQ revealed significant differences across the three groups, with chronic users reporting significantly more failures than non-users and experimenters. A series of ANCOVAs indicated that group differences in retrospective memory and use of alcohol and other drugs were driving this effect, since controlling for these variables eliminated the effect.

Our failure to find evidence for cannabis-related objective deficits on any of the prospective memory tests contrasts with previous research that has revealed such deficits [9], [10], and at first glance it appears to conflict with our findings of self-reported failures. However, the effects on the subjective measures were largely mediated by self-reported problems with retrospective memory. Given that we intentionally used objective prospective memory tests that place a heavy burden on the prospective component and minimal demands on the retrospective component of prospective memory and that effects on prospective memory appear to be mediated by problems with retrospective memory, it is not surprising that we failed to detect objective deficits. In contrast, Bartholomew et al. (2010) used tests that place minimal demands on the prospective component and increased emphases on the retrospective component [9]. Thus, it may be that cannabis users have more trouble recalling what task needs to be executed rather than recalling that a task requires execution.

As the completion of a self-report scale assessing previous experience requires the use of retrospective memory, it is possible that problems with retrospective memory interfere with cannabis users' ability to remember and rate the frequency of previous prospective memory failures. Moreover it is possible that effects are more readily apparent on subjective measures than objective measures because of the confounding influence of the acute effects of cannabis on the subjective measures. That is, some of the reported failures by cannabis users may be primarily experienced when they are under the influence of the drug. To date, the influence of acute cannabis intoxication on prospective memory has not been examined but would certainly be enlightening.

Our failure to reveal evidence for an objective deficit in prospective memory may also be related to diminished sensitivity associated with these tests. Many prospective memory tests require only one trial, while most retrospective memory tests and self-report scales contain numerous trials and items, respectively. As a result, objective prospective memory tests tend to be less sensitive than objective tests of retrospective memory and subjective measures of both domains of memory. While we attempted to increase sensitivity using the Fruit Prospective Memory Test, which required four trials, it is possible that diminished sensitivity associated with the objective prospective memory tests contributed to our failure to detect effects of chronic cannabis use on these tests. Future research should aim to use more habitual tests of prospective memory that contain an increased number of trials.

Frequent long-term cannabis use can elicit alterations in cognitive functioning that cumulate with years of use [46]. Our samples were relatively young on average, and thus may not have had a sufficient number of years of experience with cannabis for it to produce a substantial effect on prospective memory. It is also possible that the students in our sample did not experience the typical negative effects of cannabis on cognition, and that is why they were able to gain admittance to, and remain enrolled in, a fairly demanding university program. However, the estimated verbal IQ scores calculated in the second study suggest that the students were of average intelligence overall. The deficit we detected on the AVLT indicates that our cannabis users did exhibit the typical problems with retrospective memory, and the high proportion of MSI X scores indicative of a high risk of a cannabis abuse or dependence disorder suggest that enough cannabis was being used to cause significant problems in the chronic users' lives. Moreover, all of the previous investigations of the link between cannabis use and objective prospective memory have utilized similar undergraduate student samples with equivocal results [9], [10], [13]. Nevertheless, it is important that future research investigates the impact of long-term heavy cannabis use on prospective memory in an older and more representative sample.

While the present studies show that cannabis use demonstrates only small to moderate sized effects on self-reported problems with prospective memory and that these effects are driven by problems with retrospective memory, problems with alcohol, or use of other drugs it is possible that other variables play a role in the relationships we detected as well. In order to increase external validity we used few exclusion criteria when recruiting participants for our studies. However increases in external validity typically come at the expense of internal validity and it is possible that other variables associated with cannabis use also play a role in the relationships we detected between cannabis use and prospective memory. In other words, our failure to fully characterize our sample leaves open the possibility that other confounding variables are at play. For instance, family history of substance use disorders, neurological disorders, and other psychiatric disorders are known to be related to cannabis use [47]–[52] and may influence prospective memory [53]–[55]. Alternatively, each of these factors are known to influence retrospective memory [56]–[59] and thus, the mediating effects of retrospective memory may be at least partially driven by some of these variables.

In summary, our findings indicate that cannabis use, particularly problems with cannabis use, has a small to moderate sized relationship with self-reported failures of prospective memory in everyday life. However, the results of our mediation analyses and our failure to detect deficits on objective tests that minimized the burden on the retrospective component of prospective memory suggest that a primary problem with retrospective memory underlies these relationships.
